# OLDER ADULTS' PERCEPTIONS OF CANNABIS AND OPIOIDS USE

**DOI:** 10.1093/geroni/igac059.393

**Published:** 2022-12-20

**Authors:** Julie Bobitt

**Affiliations:** University of Illinois at Chicago, Chicago, Illinois, United States

## Abstract

Older adults in the U.S. are increasingly using cannabis as a method to manage pain. Some studies have linked increased cannabis use with an increase in prescription opioid use while others have suggested that older adults may be using cannabis as a way reduce/replace opioids. From April 2018 - January 2019 we conducted 12 focus groups throughout Illinois with 82 cannabis users aged 60+. To examine the relationship between cannabis and opioid use we used an inductive thematic analysis to code and theme the focus group transcripts. We found three themes 1) medical culture around opioids influences cannabis use; 2) past negative experiences with opioids influences cannabis use, and 3) aversion to ever trying opioids out of fear of an anticipated harm that may be brought about by opioid use such as overdose. In this session we present these findings and discuss cannabis use relative to opioids by older adults.

